# Analysis of the clinical relevance of antimitochondrial antibodies to the β- and γ-subunits of the F_1_F_0_-ATPase in patients with primary biliary cirrhosis

**DOI:** 10.1186/1471-230X-12-152

**Published:** 2012-10-24

**Authors:** Dominik Nann, Christoph P Berg, Beate E Preuß, Reinhild Klein

**Affiliations:** 1 Department of Internal Medicine II, University of Tuebingen, Tuebingen, Germany; 2 Department of Internal Medicine I, University of Tuebingen, Tuebingen, Germany

**Keywords:** Primary biliary cirrhosis, Antimitochondrial antibodies, Anti-M2/ODC, F_1_F_0_-ATPase, β-subunit, γ-subunit

## Abstract

**Background:**

In a recent study we showed that in patients with primary biliary cirrhosis (PBC) being positive or negative for anti-M2 antibodies reacting with the 2-oxoacid-dehydrogenase complex (ODC) also antibodies to the beta- and gamma-subunits of F_1_F_0_-ATPase (anti-β, anti-γ) occur. This is a mitochondrial enzyme but parts are also expressed on plasma membranes of endothelial cells. Here we wanted to analyse in more detail their clinical relevance.

**Methods:**

Fifty-nine untreated and histologically defined PBC patients who had been followed for at least five years were included into the study (51 anti-M2 positive, 8 anti-M2 negative). Twenty-three of them were treated in the follow up with ursodeoxycholic acid (UDCA), eight received during a trial methotrexate (MTX). In 13 patients orthotopic liver transplantation (OLT) had to be performed. Serum samples before and during therapy were available. Patients were analysed with respect to laboratory parameters, disease activity and histological stages.

Patients’ sera were tested by ELISA for IgG- and IgM-antibodies against the beta- and gamma-subunits which had been recombinant expressed in *E.coli* and highly purified by electro-elution from SDS-gels after electrophoresis.

**Results:**

Fifty-nine percent of the anti-M2 positive and 50% of the anti-M2 negative PBC patients had anti-β- and/or anti-γ-antibodies. There were no differences between anti-β- and/or anti-γ-antibody positive or negative patients with respect to biochemical parameters, immunoglobulins, histological stages or disease activity. Antibody reactivity significantly decreased during UDCA and MTX-treatment and also after OLT.

**Conclusions:**

Antibodies to the β- and γ-subunits of F_1_F_0_-ATPase occur in anti-M2 positive and –negative PBC but do not have any relevance with respect to clinical activity or prognosis. However, in contrast to the anti-M2 antibodies they decrease during UDCA and immunosuppressive therapy.

## Background

Antimitochondrial antibodies are the hallmark in the diagnosis of primary biliary cirrhosis (PBC) [1]. Several subtypes have been described [2], the most relevant being the anti-M2 antibodies directed against the five subunits of the 2-oxoacid-dehydrogenase complex (ODC), the E2- and E3-subunits of the pyruvate dehydrogenase complex (PDC) (M2a, b), the E2-subunits of the 2-oxoglutarate deyhdrogenase complex (OGDC) and the branched-chain 2-oxo-acid dehydrogenase complex (BCOADC) (M2c) and the E1 alpha- and beta-subunits of the PDC (M2d, e) [3-7]. About 95% of PBC sera react with these subunits using either the M2-antigen prepared from bovine heart mitochondria or a fusion protein consisting of the E2-subunits of ODC, the most important components [1,3,8-10]. However, in recent years we observed in an increasing incidence the presence of AMA as determined in the immunofluorescence test in PBC sera which did not react with any of the subunits of the ODC in ELISA or Western blotting [11]. In these sera we found antibodies recognising another mitochondrial inner membrane enzyme namely the F_1_F_0_-ATPase, especially its subunits beta (anti-β) and gamma (anti-γ) [11,12]. Thus, 67% of anti-M2 negative PBC patients had anti-β- and/or anti-γ-antibodies. Further analyses revealed that they occur also in anti-M2 positive PBC in about 50%. However, it turned out that they may be rather marker antibodies for an autoimmune process in the liver in general than specifically for PBC [12]. Despite this we wanted to see whether these antibodies may correlate with any specific clinical or laboratory parameters in PBC, may be indicative for an association with another (autoimmune) liver process or are influenced by different therapeutic regimes. Therefore, the present study was undertaken analysing PBC patients who had been followed for five to 30 years.

## Patients and Methods

### Patients

Fifty-nine patients with clinically and histologically defined PBC were included into the study (56 females, 3 males; mean age at time of diagnosis 48.7 years, range 19–63 years). Only patients who had been followed for at least 5 years (range up to 30 years) at the Department of Internal Medicine I were analysed. From each patient 2–30 consecutive serum samples were available (in total 425 samples, median 4 samples).

Detailed clinical, biochemical, and serological data of these patients are given in Table 1. Of the 59 patients, 54 (92%) had AMA in the immunofluorescence test (IFT), 51 (86%) reacted with the M2-antigen containing the five subunits of the ODC in the ELISA; i.e. 8 patients were anti-M2 negative, and this was confirmed by testing them by Western blotting against the recombinant ODC-subunits. Nevertheless, five of them showed the typical AMA-pattern in the IFT.

**Table 1:** Clinical, histological and serological parameters in 59 untreated patients with PBC at time of first diagnosis

**Parameters**	**Total**	**Inactive PBC**	**Active PBC**
	**n = 59**	**n = 37**	**n = 22**
Females (number)	56	36	20
Males (number)	3	1	2
**Biochemical parameters: mean + SD (median)**			
AP (U/l) (normal < 120)	440 + 374 (309)	344 + 250 (235)	647+519 (537)^a)^
ASAT (U/l) (normal < 35)	38 + 43 (24)	34 + 48 (20)	46 + 28 (43)^b)^
ALAT (U/l) (normal < 35)	51 + 56 (26)	43 + 53 (25)	62 + 62 (42)
γGT (U/l) (normal < 50)	145 + 107 (129)	128 + 86 (127)	194 + 146 (164)
bilirubin (mg%) (normal < 1.5)	1,4 + 1,8 (1,0)	0,8 + 0,7 (0,9)	2,3 + 2,8 (1,5)^b)^
IgG-globulins (mg%) (normal < 1800)	1527 + 477 (1450)	1395 + 603 (1310)	1331 + 733 (1450)
IgM-globulins (mg%) (normal < 280)	373 + 201 (324)	298 + 162 (162)	398 + 290 (427)
**Histological stages (number):**			
PBC I/II	45	37	8
PBC III/IV	14	0	14
**Autoantibodies to: number (%)**			
mitochondria (IFT)	54 (92)	33 (89)	21 (95)
M2 (ELISA)	51 (86)	31 (84)	20 (91)
nuclear dots (sp100; IFT)	14 (24)	9 (24)	5 (23)
nuclear membrane (gp210; IFT)	4 (7)	1 (3)	3 (14)
centromeres	6 (10)	2 (5)	3 (14)

Significance between patients with inactive and active PBC: ^a)^ trend with p = 0.08; ^b)^ p < 0.05.

Forty-five patients were at time of first diagnosis in stage I/II, 14 in stage III/IV. Furthermore, the patients were divided into two groups according to their clinical course (active vs inactive): Group A (active course) consisted of 22 patients, who were at time of first diagnosis in late stages or who were in stage I/II but developed within 5–10 years signs of liver cirrhosis (histologically development of stage III/IV, hyperbilirubinemia, portal hypertension, necessity of liver transplantation, death because of liver failure); group B (inactive course) included 37 patients who were at first diagnosis in stage I/II and did not develop any signs of disease progression. Biochemical parameters of these two groups are given in Table 1. Both groups differed significantly with respect to ASAT and bilirubin levels, other biochemical parameters were similar (Table 1).

None of these patients received any therapy at first presentation at our department. Eight of them were treated transiently with methotrexate (15–20 mg/week) in the course of a trial in the time between 1988 and 1989 when no other forms of therapy were available. Beyond 1994 all patients received ursodeoxycholic acid (UDCA; 1000–1500 mg/day).

In 13 patients orthotopic liver transplantation had to be performed. From these patients serum samples at different time points after transplantation were available (0–6 months: n = 5; 6–12 months: n = 7; 12–24 months: n = 5; > 24 months: n = 9).

As controls, sera from 41 healthy blood donors were used (kindly provided by Prof. D. Wernet, Department of Transfusion Medicine, University of Tuebingen).

Patients’ sera were stored at −20°C.

Sera from all patients had been taken for diagnostic purposes. The study was approved by the local ethic committee of the University Hospital Tuebingen.

## Methods

### Antigens and cell lines

For the immunofluorescence test (IFT) cryostat sections from rat liver, kidney, stomach and heart were used [13] as well as Chang liver cells for the demonstration of distinct antinuclear antibody (ANA) patterns.

The M2-antigen known to containing all five determinants corresponding to the 2-oxoacid dehydrogenase complex [3,6] was prepared from bovine heart mitochondria by chloroform release as described by Beechey et al. [14] and Lindenborn-Fotinos et al. [15].

The recombinant PDC-E2, OGDC and BCOADC was obtained from DIARECT AG (Freiburg, Germany).

The β− and γ-subunits of the F_1_F_0_-ATPase were expressed in *E.coli* as recently described [12]. For further purification and in order to avoid bacterial contaminations, the recombinant proteins were applied to SDS-gel electrophoresis; after Coomassie-Blue-staining the relevant determinants were excised and eluted as recently described [12].

### Methods for detection of autoantibodies

IFT was performed for the demonstration of AMA according to standardized methods [13] using cryostat sections from rat liver, kidney, heart, and stomach as well as human thyroid. Sera were diluted 1:10, titres > 1:40 were considered positive. Furthermore, sera were analysed by IFT on cell cultures for the demonstration of different antinuclear antibody specificities as reported [16].

Antibodies to M2, PDC-E2, OGDC and BCOADC were detected by ELISA and Western blotting as previously described [6,11,12]. Anti-β and anti-γ antibodies were determined only by ELISA because Western blotting seems to be not a suitable method [12]. Briefly, The highly purified recombinant β- and γ-subunits obtained by electro-elution from the SDS-gels were used for coating the microtiter plates at a concentration of 8 μg/ml, patients’ sera were diluted 1:500. For the visualization of bound autoantibodies, polyvalent peroxidase conjugated goat anti-human IgG- and IgM-antibodies were used in parallel (IgG: dilution 1:3000, IgM: dilution 1:2000; Dianova, Hamburg, Germany), as substrate o-phenylenediamine was applied. Results were given as absorbance multiplied by 1000.

Optimal antigen- and serum concentrations had been evaluated by serial dilutions prior to the study.

Normal ranges for antibody reactivities with the antigens were determined by analysis of sera from 41 healthy blood donors. Mean values of their absorbance (× 1000) plus twice the standard deviation were defined as cut-off values, and these cut-off values were also confirmed by ROC (receiver operating curve) analysis (Additional file 1: Figure S1).

### Statistics

Statistical analysis was performed with SSPS 19 using the non parametric Mann–Whitney test for unpaired and the Wilcoxon-test for paired groups. For the comparison of prevalences, the Chi square test was applied.

## Results

### Prevalence and reactivity of anti-β- and γ-antibodies in 59 untreated PBC-patients at time of first presentation

Thirty-four (58%) of the 59 PBC patients had either anti-β- or anti-γ-antibodies or both (Table 2). The antibodies were of the IgG- as well as of the IgM-type (Table 3).

**Table 2:** Prevalence of antibodies to the β- and γ-subunits in anti-M2 positive and –negative PBC

**Antibodies to the**		**Anti-M2**	**Anti-M2**	**Total**
		**positive (n = 51)**	**negative (n = 8)**	**(n = 59)**
β−subunit	γ−subunit	Number (%)		
+	+	14 (27)	3 (38)	17 (29)
+	-	2 (4)	0	2 (3)
-	+	14 (27)	1 (13)	15 (25)
-	-	21 (41)	4 (50)	25 (42)

Analysis of 59 patients.

**Table 3:** Immunoglobulin classes of antibodies to the β- and γ-subunit

**Antibodies to the**	**Ig-class**	**Anti-M2**	**Anti-M2 negative**	**Total**
		**positive (n = 51)**	**(n = 8)**	**(n = 59)**
		**number (%) positive**		
β−subunit	IgG	11 (22)	1 (13)	12 (20)
	IgM	9 (18)	2 (25)	11 (19)
γ−subunit	IgG	16 (35)	2 (25)	18 (31)
	IgM	23 (45)	3 (38)	26 (44)

Analysis of 59 patients with anti-M2 positive and –negative PBC.

**Figure 1:**
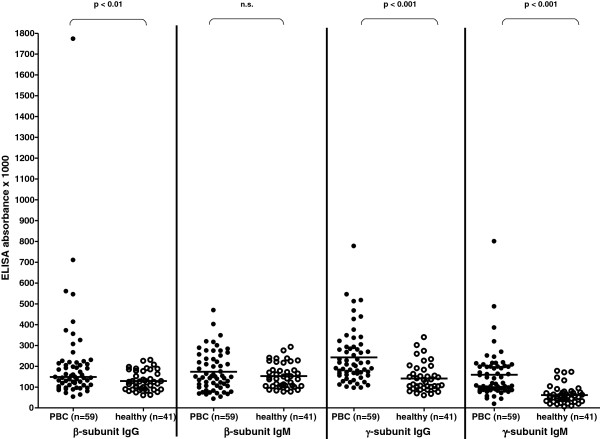
**Reactivity of antibodies to the β− and γ−subunits of F**_**1**_**F**_**0**_**-ATPase in PBC-patients and healthy controls.** Sera from 59 PBC patients and 41 healthy individuals were analysed. Individual values are given. **-** = median.

IgG antibody activity towards the β-subunit was significantly higher in PBC patients as compared to healthy controls, while IgM reactivity did not differ between both groups (Figure 1). In contrast, both, IgG- and IgM-antibody reactivity to the γ-subunit was significantly higher in the PBC patients than in the healthy individuals.

### Correlation of the different types of antimitochondrial antibodies in 59 untreated PBC-patients at time of first presentation

As demonstrated in Table 1, 92% of the 59 patients showed the typical AMA-pattern in the IFT, but only 51 (86%) reacted with the M2-antigen, the remaining eight patients were negative for all five subunits of the ODC. Five of them had AMA in the IFT, two had antibodies to nuclear dots (sp100), two to centromeres, and none to nuclear membrane (gp210). Of the eight anti-M2/ODC negative patients, 4 (50%) had antibodies to the β- and/or γ-subunit (Table 2). Comparing the reactivity of anti-β- and -γ−antibodies in patients with anti-M2 positive and –negative PBC, there was no statistically significant difference between both groups (data not shown).

There was no correlation between antibody reactivity towards M2/ODC and the β-subunit for IgG or IgM-antibodies (r = 0.0002 and −0.03, respectively) or the γ-subunit (r = 0.35 for IgG and r = −0.01 for IgM antibodies). Fortyeight of the 59 sera (81%) reacted in the Western blot with the M2a-determinant corresponding to the E2-subunit of the PDC, but again there was no correlation to the presence of anti-β- or anti-γ antibodies of the IgG- or IgM-type (data not shown).

### Correlation of anti-β- and -γ-antibodies with clinical and histological parameters

**Figure 2:**
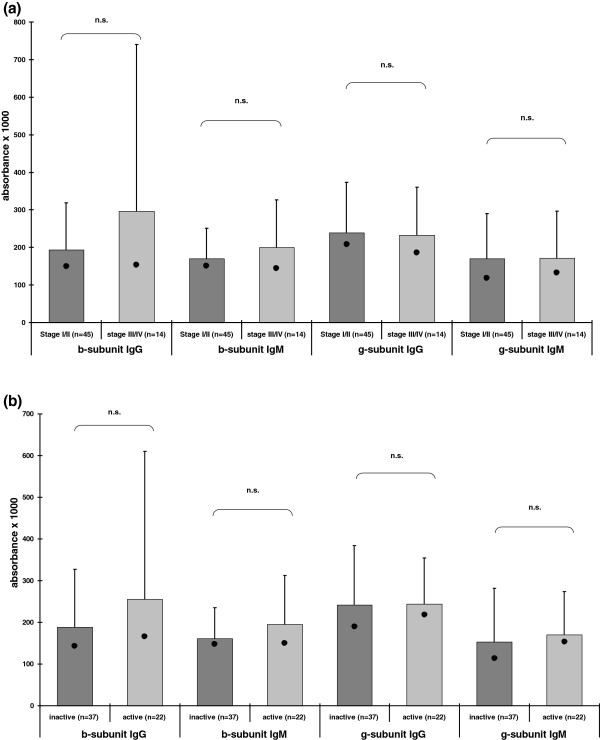
**Correlation of antibody reactivity to the β− and γ−subunit with histological (a) and clinical parameters (b).** Data are shown as means (bars) plus standard deviation and median (·).

There was no correlation of the presence or absence or reactivitiy of antibodies to the β− and γ−subunit with histological stages (Figure 2a, Table 4). Comparing patients with an inactive and those with an active form of PBC as defined in ‘patients’, again no significant differences were observed in prevalence or reactivity of anti-β− and/or-γ−antibodies (Figure 2b, Table 4).

**Table 4:** Correlation of antibodies to the β− or γ−subunit with histological stages and disease activity of PBC

		**Histological stages**		**Disease activity**	
**Antibodies to the**		**I/II (n = 45)**	**III/IV (n = 14)**	**Inactive (n = 37)**	**Active (n = 22)**
β−subunit	γ−subunit	Number (%)			
+	+	12 (27)	5 (36)	10 (27)	7 (32)
+	-	1 (2)	1 (7)	1 (3)	1 (5)
-	+	13 (29)	3 (21)	10 (27)	6 (27)
-	-	19 (42)	5 (36)	16 (43)	8 (36)

Analysis of 59 patients.

Also biochemical parameters (AP, γGT, ASAT, ALAT, bilirubin, IgG-, IgA-, IgM-globulins) did not correlate with antibodies to the β− or γ−subunit (not shown).

### Influence of treatment on anti-β- and γ-antibodies

**Figure 3:**
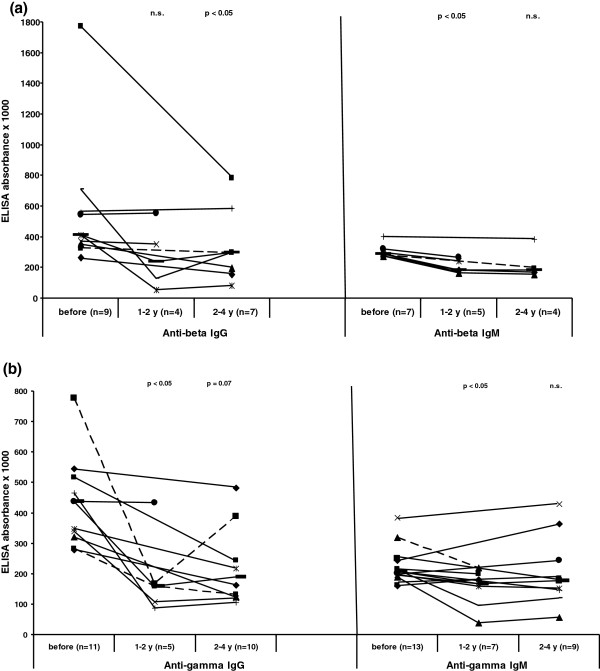
**Influence of UDCA therapy on reactivity of anti-β (a) and –γ−antibodies (b).** Only patients being positive for the respective subunits before therapy are included. Individual values at the three time points are given. **-** = median.

In a further step we wanted to see whether the antibodies to the β− and γ−subunits are influenced by UDCA therapy. In this analysis 23 patients were included, of whom exact data on beginning of the therapy, dosage and compliance were available and who were treated for at least one year. From 11 of them sera were available within the time interval one to two years and from 19 patients within the interval two to four years after starting the therapy. Seventeen of the 23 patients (74%) had anti-β− and/or –γ−antibodies before treatment. The prevalence significantly decreased to 45% (p < 0.01) being positive after 1–2 and 58% (p < 0.05) after 2–4 year treatment. There was also evidence for a decrease of anti-β- and anti-γ reactivity (absorbance x 1000) during UDCA treatment in those patients being positive before therapy, which reached statistical significance despite the low number of patients (Figure 3a, b).

In contrast, the prevalence and reactivity of anti-M2 antibodies was not influenced by this kind of treatment (not shown).

Eight patients were also treated with methotrexate for at least 5 months (range 5 months-13 years, median 14 months). Serum samples before treatment and at the last time point during therapy were analysed. Five of these patients (63%) had at least one of the two antibodies before therapy; at the last time point during therapy only 38% were still positive. Performing statistical analysis in all 8 patients independently whether they were anti-β- or –γ-positive before MTX-therapy or not, there was a significant decrease in reactivity (absorbance × 1000) during therapy (anti-β IgG: before therapy absorbance × 1000: mean ± SD: 139 ± 26; median: 140; during therapy: 108 ± 28; median: 100; p < 0.05; anti-β IgM: before therapy: 166 ± 73; median: 136; during therapy: 131 ± 36; median: 132; p < 0.05; anti-γ IgG before therapy: 248 ± 93; median: 223; during therapy: 171 ± 51; median: 156; p < 0.05; anti-γ IgM: before therapy: 149 ± 84; median 106; during therapy: 106 ± 53; median 91; p < 0.05). However, analysing only those patients being positive before MTX-therapy, statistical analysis was not possible due to the low number of patients.

In contrast, prevalence and reactivity of antibodies to ODC (M2) were not significantly influenced by this kind of treatment (IgG-antibodies: before therapy absorbance x1000: mean ± SD: 680 ± 425; median 769; during therapy: 750 ± 456; median 845; IgM-antibodies: before therapy 685 ± 411; median 535; during therapy: 690 ± 353; median 575).

### Anti-β- and-γantibody reactivity after OLT

**Figure 4:**
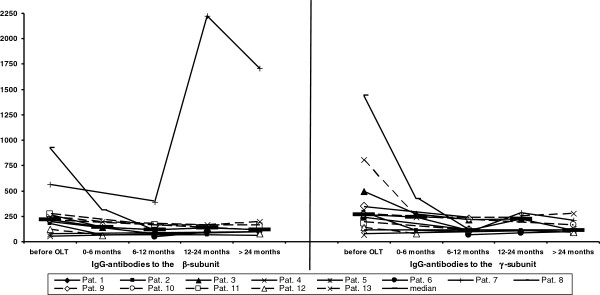
**IgG-antibody reactivity of anti−β− and –γ−antibodies in 13 PBC patients before and after liver transplantation.** Individual values at the different time points are given. **-** = median.

In 13 patients liver transplantation had to be performed. Nine of these patients had anti-β− and/or –γ−antibodies before transplantation. The prevalence of IgG antibodies and also their reactivity significantly decreased after OLT (Figure 4), while IgM antibodies were not affected. Similar data were obtained for the anti-M2 reactivity (data not shown). Interestingly, there was one patient in whom IgG reactivity to the b-subunit strongly increased after OLT, and this patient had acquired hepatitis B at that time.

## Discussion

It is well known, that antibodies to the 2-oxoaciddehydrogenase complex – previously named anti-M2 – are excellent marker antibodies for the serological diagnosis of PBC but that they do not correlate with any clinical, histological or laboratory parameters, and that they are not influenced by UDCA treatment and only transiently decrease after liver transplantation [17]. Recently we described a further AMA-type in PBC reacting with the β− and γ−subunits of the inner mitochondrial membrane enzyme F_1_F_0_-ATPase [11,12]. However, these antibodies were not restricted to PBC but also found in patients with autoimmune (but not viral) hepatitis and primary sclerosing cholangitis implying that they may be a marker for an autoimmune liver process in general. We, therefore, wanted to see whether this novel antibody type may be indicative for distinct clinical subtypes within the PBC patients, i.e. whether they may point towards an association with another liver process. Analysing a large group of PBC patients followed for a long time period we observed, however, no correlation of anti-β− and γ−antibodies with histological stages, disease activity, any laboratory parameters, or an association with other autoimmune processes, i.e. in this respect they resemble the behaviour of anti-ODC/M2 antibodies. But in contrast to the anti-M2 antibodies, the anti-β− and γ−antibodies decreased during treatment with UDCA and also during immunosuppressive therapy as shown in the present study for MTX – a phenomenon which is otherwise typical for autoantibodies in autoimmune hepatitis.

The treatment of eight patients with MTX had been designed in a period when uncontrolled studies suggested that it may be effective in precirrhotic PBC [18-22], but this is still controversially discussed [23,24]. The number of our patients treated with MTX was too low to allow any conclusions with respect to its clinical efficacy; nevertheless it was of interest that despite this low number of patients the reactivities towards the β− and γ−subunits significantly decreased.

IgG antibodies to the β− and γ−subunit also decreased after liver transplantation – similarly to anti-M2 [17] - but it has still to be evaluated in a larger group of patients followed for several years whether this effect is only transient.

We also confirmed that anti-β− and/or γ−antibodies occur in about 50% of anti-M2 negative PBC [11], and only some of them had AMA in the IFT; i.e. antibodies to F1-ATPase are not closely related with a positive IFT pattern as recently pointed out [11,12]. Hidden epitopes may be responsible for this phenomenon using cryostat sections in the IFT. Further, antibodies to the F1F0-ATPase subunits did not correlate with anti-M2/ODC reactivity or with the presence or absence of PBC specific antibodies to nuclear antigens such as gp210, sp100 or centromeres [25], indicating that they are an independent phenomenon.

The induction of antibodies to F_1_F_0_-ATPase remains still obscure; however, it has been shown that the F_1_F_0_-ATPase is expressed on the cell surface of endothelial cells, adipocytes, hepatocytes, and tumor cells, the F_1_-domain facing outside [26-30]. Furthermore, components of the F_1_F_0_-ATPase, usually the β-chain, have been characterized as cell surface receptors for different ligands involved in intracellular pH homeostasis, angiogenesis, and lipid metabolites. Thus, apoA-I binds to F_0_F_1_-ATPase expressed on endothelial cells and stimulates proliferation of endothelial cells by promoting extracellular ATP production [31]. Moreover, angiostatin, an endogenous angiogenesis inhibitor also binds to cell surface F_1_F_0_-ATPase on endothelial cells acting as an inhibitor of cell proliferation [30,32]. It is, therefore, conceivable, that this plasma-membrane-exposed F_1_F_0_-ATPase may become a neoantigen by binding of abnormal ligands hereby developing into target for the immune response. In this respect our preliminary observations are of interest indicating that also CD8+ T cells recognizing especially the β-subunit exist, and that at least the β-subunit seems to be expressed by cholangiocytes (unpublished observations). Interestingly, there was one patient in whom IgG reactivity to the b-subunit strongly increased after OLT in close relationship to the acquisition of viral hepatitis B supporting the hypothesis of neoantigen formation, probably induced by an infection. On the other hand, testing a large group of patients with viral hepatitis (n = 110), only 12 had IgG anti-beta-antibodies [12].

## Conclusions

In conclusion we have shown that the antibodies to the β− or γ−subunits of the F_1_F_0_-ATPase found in autoimmune liver disorders in general do not have a distinct importance in patients with PBC with respect to clinical, laboratory or histological parameters or prognosis. Although their clinical relevance is, therefore, at this moment limited, these antibodies add to the plethora of autoantibodies in PBC again underlining that its pathogenesis is not only PDC-E2 related. Quite on the contrary, also the F_1_F_0_-ATPase has to be taken into consideration as a target antigen considering the fact that it is not any more merely a mitochondrial enzyme but also exposed on plasma membranes serving as a receptor for various ligands.

## Abbreviations

AIH: autoimmune hepatitis; AMA: antimitochondrial antibodies; ANA: antinuclear antibodies; ALAT: alanine aminotransferase; ASAT: aspartate aminotransferase; AP: alkaline phosphatase; ATP: Adenosinetriphosphate; BCOADC: branched-chain 2-oxo-acid dehydrogenase complex; ELISA: enzyme linked immunosorbent assay; γGT: gamma-glutamyltransferase; IFT: immunofluorescence test; Ig: immunoglobulin; MTX: methotrexate; ODC: 2-oxoacid-dehydrogenase complex; OGDC: 2-oxoglutarate dehydrogenase complex; OLT: orthotopic liver transplantation; PBC: primary biliary cirrhosis; PSC: primary sclerosing cholangitis; PDC: pyruvate dehydrogenase; SDS: sodiumdodecylsulfate; UDCA: ursodeoxycholic acid.

## Competing interests

None of the authors has to declare any financial or non-financial interests.

## Authors’ contributions

DN and BEP performed the experiments. CPB provided patients’ material and clinical data. CPB and RK designed the research, DN and RK analyzed the data and wrote the paper. All authors read and approved the final manuscript.

## Pre-publication history

The pre-publication history for this paper can be accessed here:

http://www.biomedcentral.com/1471-230X/12/152/prepub

## Supplementary Material

Additional file 1 **Figure S1.** ROC curves for IgG- and IgM antibodies to the β- and γ-subunit of F_1_F_0_-ATPase analysed with 59 sera from PBC patients without therapy and sera from 41 healthy controls. Click here for file
