# Supplementary material for: Analysis of the clinical relevance of antimitochondrial antibodies to the β- and γ-subunits of the F1F0-ATPase in patients with primary biliary cirrhosis
Source: BMC Gastroenterol. 2012 Oct 24;12:152. doi: 10.1186/1471-230X-12-152 (PMC3523002; doi:10.1186/1471-230X-12-152)

**Supplementary material**

**Figure legend**

ROC curves for IgG- and IgM antibodies to the β- and γ-subunit of F_1_F_0_-ATPase analysed with 59 sera from PBC patients without therapy and sera from 41 healthy controls.


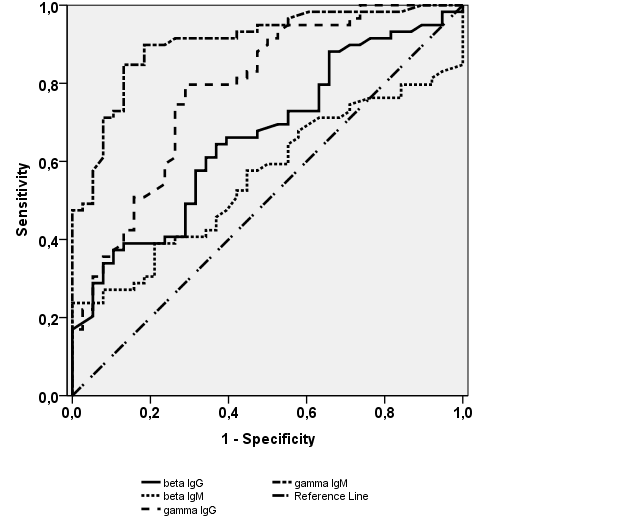

Supplement: Additional file 1 — Figure S1. ROC curves for IgG- and IgM antibodies to the β- and γ-subunit of F1F0-ATPase analysed with 59 sera from PBC patients without therapy and sera from 41 healthy controls. [file 1471-230X-12-152-S1.doc]
